# Evidence for a Prehypertensive Water Dysregulation Affecting the Development of Hypertension: Results of Very Early Treatment of Vasopressin V1 and V2 Antagonism in Spontaneously Hypertensive Rats

**DOI:** 10.3389/fcvm.2022.897244

**Published:** 2022-06-01

**Authors:** Ignazio Verzicco, Stefano Tedeschi, Gallia Graiani, Alice Bongrani, Maria Luisa Carnevali, Simona Dancelli, Jessica Zappa, Silvia Mattei, Achiropita Bovino, Stefania Cavazzini, Rossana Rocco, Anna Calvi, Barbara Palladini, Riccardo Volpi, Valentina Cannone, Pietro Coghi, Alberico Borghetti, Aderville Cabassi

**Affiliations:** ^1^Cardiorenal and Hypertension Research Unit, Physiopathology Unit, Clinica Medica Generale e Terapia Medica, Department of Medicine and Surgery (DIMEC), University of Parma, Parma, Italy; ^2^Histology and Histopathology Unit and Molecular Biology Laboratory, Dental School Parma, University of Parma, Parma, Italy; ^3^Nefrologia e Dialisi, Azienda USL – Istituto di Ricerca a Carattere Scientifico IRCCS Reggio Emilia, Reggio Emilia, Italy; ^4^Internal Medicine Unit, Ospedale Fidenza, Azienda USL Parma, Parma, Italy; ^5^Laboratory of Industrial Toxicology, DIMEC, University of Parma, Parma, Italy

**Keywords:** aquaporin 2, experimental hypertension, pre-hypertensive phase, spontaneously hypertensive rat, vasopressin, vasopressin receptor 1 and 2 antagonism

## Abstract

In addition to long-term regulation of blood pressure (BP), in the kidney resides the initial trigger for hypertension development due to an altered capacity to excrete sodium and water. Betaine is one of the major organic osmolytes, and its betaine/gamma-aminobutyric acid transporter (BGT-1) expression in the renal medulla relates to interstitial tonicity and urinary osmolality and volume. This study investigated altered water and sodium balance as well as changes in antidiuretic hormone (ADH) activity in female spontaneously hypertensive (SHR) and normotensive Wistar Kyoto (WKY) rats from their 3–5 weeks of age (prehypertensive phase) to SHR’s 28–30 weeks of age (established hypertension-organ damage). Young prehypertensive SHRs showed a reduced daily urine output, an elevated urine osmolarity, and higher immunostaining of tubule BGT-1, alpha-1-Na-K ATPase in the outer medulla vs. age-matched WKY. ADH circulating levels were not different between young prehypertensive SHR and WKY, but the urine aquaporin2 (AQP2)/creatinine ratio and labeling of AQP2 in the collecting duct were increased. At 28–30 weeks, hypertensive SHR with moderate renal failure did not show any difference in urinary osmolarity, urine AQP2/creatinine ratio, tubule BGT-1, and alpha-1-Na-K ATPase as compared with WKY. These results suggest an increased sensitivity to ADH in prehypertensive female SHR. On this basis, a second series of experiments were set to study the role of ADH V1 and V2 receptors in the development of hypertension, and a group of female prehypertensive SHRs were treated from the 25th to 49th day of age with either V1 (OPC21268) or V2 (OPC 41061) receptor antagonists to evaluate the BP time course. OPC 41061-treated SHRs had a delayed development of hypertension for 5 weeks without effect in OPC 21268-treated SHRs. In prehypertensive female SHR, an increased renal ADH sensitivity is crucial for the development of hypertension by favoring a positive water balance. Early treatment with selective V2 antagonism delays future hypertension development in young SHRs.

## Introduction

Sodium and water homeostasis is essential for the development and the long-term regulation of blood pressure (BP), and the kidney is a major player in this process (1, 2). Since Guyton’s hypothesis, based on a two-compartment model where the increase of intravascular fluid volume related to impaired kidney sodium excretion is responsible for BP rise, the defective renal sodium excretion and altered pressure-natriuresis mechanism represent the basis for initiating and maintaining arterial hypertension. The deviation of the pressure-natriuresis mechanism indicates that arterial hypertension is necessary to balance the renal natriuretic inefficiency (3, 4). Kidney cross-transplantation studies support this hypothesis, showing that normotensive rats become hypertensive when they receive a kidney from a hypertensive donor (5–7). The two-compartment sodium model has been implemented, including a third compartment of tissues of non-osmotically active sodium storage (8), mainly the skin that supports a new sodium-mediated immune system activation as a BP regulator (9). Despite at least four decades of extensive research (10), the role of arginine-vasopressin (known as antidiuretic hormone, ADH) and water balance dysregulation in the development and maintenance of arterial hypertension remains controversial.

### ADH Physiology and Water Balance

The nonapeptide ADH is synthesized by the hypothalamic paraventricular and supraoptic nuclei and is stored and released from the posterior pituitary gland under a serum osmolality rise, a relevant volume depletion (> 7%), and a circadian rhythmicity (10, 11). Once released into the bloodstream, ADH has a very short half-life (around 20 min) and can bind to platelets, making its plasma measurements highly variable (12); it exerts its functions *via* G-protein–coupled receptors V1 (a and b subtypes) and V2 receptors. V1a receptor induces vasoconstriction in most of the vascular beds; it is expressed in vascular smooth muscle cells, cardiac myocytes, kidney vasa recta, and medullary interstitial cells; and it participates in the regulation of glucocorticoids by inducing the release of adrenocorticotropic hormone from the anterior pituitary gland (10). V2 receptor activation induces water absorption and sodium retention through aquaporin 2 (AQP2) gene expression stimulation and protein translocation to the apical membrane of the principal cells of the kidney collecting duct (11, 13). The process of AQP2-bearing vesicles docking and fusion to the apical plasma membrane as well as the exocytosis/endocytosis balance are tightly regulated by ADH through posttranslational modifications such as phosphorylation, ubiquitylation, and degradation (14); other factors such as prostaglandin E2, estrogen, and interstitial medullary hypertonicity *per se* can also affect AQP2 expression and trafficking independently from ADH (15–19). In addition to direct transcellular water transport, ADH contributes to interstitial intramedullary hyperosmolality and allows gradient-driven water absorption by increasing sodium absorption in several other tubular segments [thick-ascending limb of Henle-luminal Na-K-2Cl cotransporter (NKCC2) and basolateral alpha-1-Na-K ATPase, distal convoluted tubulesNa-Cl co-transporter (NCC), and collecting ducts-epithelial sodium channel (ENaC)] and by favoring urea cycling and interstitial urea medullary absorption (increased expression of urea medullary transporters A1 and A3) (20, 21). Sodium and urea are the major interstitial medullary osmolytes, whereas organic osmolytes in renal intracellular fluid to balance the hypertonic interstitium include polyols (sorbitol), neutral free amino acids, and the combination of urea and methylamines (22). One of the principal organic osmolytes is represented by betaine, a neutral free amino acid, and a methylamine. Betaine is synthesized by proximal tubule cells and transported by a specific transporter, the betaine/GABA transporter 1 (BTG-1) to the medulla. Interstitial hypertonicity causes an increase in basolateral membrane BGT-1, which couples the transport of betaine to that of chloride and sodium, allowing betaine to enter the cell from the extracellular fluid. Under normotonic conditions, BGT-1, which is mainly located in the cytoplasm, translocates to basolateral plasmalemmas when interstitial tonicity increases. Intracellular accumulation of betaine and the higher expression of BGT-1 in the tubule reflect the higher interstitial tonicity that increases urine osmolarity in the presence of ADH.

### ADH Contribution to Hypertension

Contrasting results on ADH plasma and urinary levels have been reported in experimental models of hypertension as well as in human essential hypertension (10, 23–25). The slight increase of urinary ADH observed both in benign and malignant essential hypertensive adults and in adolescent patients does not unambiguously and explicitly indicate a relevant contribution of ADH to the development or maintenance of hypertension (26–29). Increased circulating ADH levels have also been reported in prehypertensive young and hypertensive middle-aged spontaneously hypertensive rats (SHR), the most widely used animal model of human essential hypertension (30), as compared to their normotensive controls Wistar Kyoto (WKY), but not in the SHR-stroke-prone strain, where ADH levels were markedly reduced (31, 32). Discordant results on neuron ADH content have been reported as well in this strain: cultured brain neurons from newborn SHR (1-day-old) contained fewer ADH levels than age-matched WKY, as in paraventricular hypothalamic neurons at 6 and 12 weeks of age and in the neurons from the brain stem of adult SHR. When compared with WKY, ADH content was similar in prehypertensive 3-week-old SHR neurons from the hypothalamic paraventricular nucleus but increased in the neurohypophyseal neurons (33–36). In another model of experimental hypertension related to blood volume expansion (deoxycorticosterone-salt hypertensive rat), ADH levels were elevated not only in plasma but also in 24-h urinary excretion, whose increase paralleled the rise in BP levels (26, 37). The importance of ADH in this model was further demonstrated by the fact that hypertension development is accelerated by treating the rats with small doses of argipressin (Pitressin) and that surgically induced diabetes insipidus prevents the development of hypertension (38). Another observation supporting the role of ADH in the development of hypertension in this model relates the fact that Brattleboro rats with hereditary hypothalamic diabetes insipidus did not develop hypertension when treated with deoxycorticosterone-salt (39). In deoxycorticosterone-salt hypertensive rat, it seems that ADH differently contributes to hypertension with a prevalent antidiuretic effect observed during the early phases of deoxycorticosterone-salt hypertension (40) and a direct vasopressor effect in the established phase of hypertension (41). Again, non-univocal results on ADH’s role in hypertension development were reported in salt-sensitive and salt resistant Dahl rats where even if the former showed increases in ADH levels, ADH receptor antagonism failed to reduce BP levels (26). Similar results were observed in the experimental model of partial nephrectomy-salt hypertension after a 70% reduction in renal mass followed by drinking a 1% NaCl solution. Treatment with a vasopressin pressor antagonist in this model had only a small effect on arterial pressure (42). On the contrary, a sustained hypertension could not be produced with the partial nephrectomy-salt protocol in rats with hereditary hypothalamic diabetes insipidus (26, 42).

Although an increased vascular sensitivity to ADH has been reported in several experimental models of hypertension, such as the spontaneously hypertensive (SHR) (43), the deoxycorticosterone (DOC)-salt hypertensive (44), the Dahl salt-sensitive hypertensive (45), the New Zealand genetically hypertensive rats (46), ADH V1a receptor-selective antagonism lowers BP in mineralocorticoid-related hypertension (47) but not in renovascular hypertension (48). Furthermore, it has been reported that chronic infusion of intravenous and renal intramedullary of a selective vasopressin V1a receptor agonist resulted in sustained hypertension, implying that the V1a receptor in the renal medulla is a mediator of the hypertensive effect (49–52); these results appear to be supported by the fact that V1a receptor knockout mice are resistance to salt-induced hypertension (53). Furthermore, while the role of ADH in the development of hypertension *via* V1a receptor stimulation was shown by the hypotensive effect of OPC-21268, a non-peptide ADH V1a antagonist, in young SHR, with a more pronounced effect in male than in female rats and the interesting observation of a persistence of low BP levels after the drug withdrawal (54), other reports were unable to demonstrate such a hypotensive effect by the chronic treatment with specific V1 receptor antagonism (55). If the evidence could indicate a certain role in the development of hypertension, this seems not to be the case in the maintenance of arterial hypertension because no effect of OPC-21268 was observed in the established hypertensive phase of adult SHR (54, 56). The involvement of V2 receptors in ADH’s potential role in the development of hypertension was also conflicting and inconclusive, observing in fact both a reduction in BP after the administration of V2 receptor antagonist administration OPC 31260 to young male SHR in the prehypertensive phase (57), but also the lack of a hypotensive effect in young SHR but a significant hypertensive effect in the adult SHR (58).

With these promises, this study investigated the abnormalities in water and sodium balance and osmoregulation in female SHRs from their prehypertensive phase until the adult age when arterial hypertension is established and organ damage has already developed. We also evaluated the effects of a short period of oral treatment with selective V1a and V2 receptor antagonists administered very early in the prehypertensive phase to measure the contribution of ADH with its pressor and aquaretic effects on the future development of arterial hypertension in SHR, the most commonly used experimental model of human essential polygenic hypertension.

## Materials and Methods

### Animals and Experimental Protocols

Rats included in this study were treated in accordance with the European Council on Animal Care guidelines and the protocols were approved by the local institutional animal ethics committee. Two experimental studies with a large number of 3-week-old female SHR (*n* = 86) and Wistar-Kyoto (WKY) rats (*n* = 58) were set up to evaluate: (i) (first protocol) the time course of water imbalances measured as ADH renal sensitivity and AQP2 expression and excretion from the prehypertensive phase to the established hypertension and organ damage development in SHR compared to age-matched WKY and (ii) (second protocol) the effect of a very early treatment with ADH V1 and V2 receptor antagonists on future development of arterial hypertension in SHR strain.

### First Protocol: Water Balance From Prehypertensive Phase to Hypertensive Organ Damage

Four batches for a total of 104 3-week-old female SHR (*n* = 58) and Wistar Kyoto (WKY, *n* = 46) rats were purchased over a period of 5 years from three different European colonies to avoid potential phenotype differences associated with breeding or alimentary factors (Charles River Laboratories, Calco, Italy; Charles River France L’Arbresle, France; Charles River Nederland, Maastricht, Netherlands). Rats were housed, fed a standard laboratory rat diet (Basal Purified Diet 5755C, 0.54% sodium chloride, Purina Mills Inc., St. Louis, MO, United States), had free access to drinking water, and were maintained on a 12-h light/dark cycle, at constant humidity (40–46%) and temperature (22–24°C) (59). After a few days of acclimatization, conscious rats from both groups underwent indirect tail-cuff measurements of systolic BP by plethysmography (Harvard Apparatus Ltd., South Natick, Massachusetts, United States) and recorded on a Maclab/8 system (AD Instruments Ltd., Castle Hill, New South Wales, Australia) as we previously described (60): four to five measurements were taken in each rat and then averaged. Young rats were then individually kept for 3 days in metabolic cages to allow daily 24-h urine collection and food and water intake measurements. The measurement of these parameters refers to the second and third days of metabolic cage stay. Urine albumin, electrolytes, creatinine, osmolality, and AQP2 were evaluated and averaged; both the urine albumin to creatinine ratio as well as the AQP2 to creatinine ratio were calculated. A quantitative sandwich enzyme immunoassay was used to evaluate urine AQP2 and was performed according to manufacturer instructions and done in triplicate (Rat Aquaporin 2 ELISA Kit, MBS703718, MBS San Diego, California, United States). The intra- and interassay coefficients of variation were 7 and 8%, respectively. At the end of the 3-day time in the metabolic cage, 23 SHR and 21 WKY rats underwent blood sampling from the inferior vena cava and basal laboratory tests, including plasma electrolytes, creatinine, and osmolality were performed. Venous blood samples for the determination of plasma ADH were collected using a prechilled heparin-coated tube and immediately centrifuged for 30 min at 3,000 *g* at 4°C; plasma samples were then stored at –80°C until assays were performed without any freeze-thaw cycles. The enzyme-linked immunosorbent assay was performed according to manufacturer instructions and was done in triplicate (Rat Vasopressin ELISA Kit, MBS3808966, MBS San Diego, California, United States). The intra- and interassay coefficients of variation were 9 and 12%, respectively, and the analytical sensitivity was 0.95 pg/ml.

### Immunohistochemistry and Immunofluorescence Studies

Following the experiment period in the metabolic cage, a group of young female SHR (*n* = 28) and age-matched WKY (*n* = 23) were euthanized with diethyl ether. As we previously described (61), the rat abdomen was opened and the vena cava cannulated with polyethylene tubing (PE-50) and perfused by a roller pump for 30 s with phosphate buffer saline, the right renal artery clamped, and the kidney was rapidly removed from its attachments (renal artery and vein, and fat and adrenal glands). After excision, the right kidney was repeatedly rinsed with 15% and 30% sucrose solutions at 4°C and then frozen embedded in OCT compound for immunofluorescence labeling of cryosections as previously described (62). The left kidney, still attached to vessels, was initially perfused by a roller pump for 5 min with a solution containing 4% of paraformaldehyde to fix the tissues and then excised and embedded in paraffin for subsequent immunohistochemical investigations (immunoperoxidase reaction) (61). After being dewaxed and rehydrated, sections (5–7 μm) underwent microwave antigen retrieval in citrate buffer (citric acid, 10 mM, pH = 6) followed by the blockade of endogenous peroxidase with hydrogen peroxide solution (3%). The sections were then incubated with primary rabbit polyclonal anti-betaine γ-amino-n-butyric acid transporter-1 (BGT-1) (Anti-BGT-1, 1:250 dilution, HPA034973, Sigma-Aldrich antibodies), anti-Aquaporin2-phospho S256 (Anti-AQP2-phospho S256, 1:200 dilution, ab111346), mouse monoclonal anti-alpha 1 Na-K ATPase (1:400 dilution, ab211130), and rabbit monoclonal anti-Uromodulin-Tamm-Horsfall (Anti-UMOD, 1:250 dilution, ab256473) (Abcam, Cambridge Biomedical Campus, Cambridge, United Kingdom; Prodotti Gianni S.p.A., Milano, Italy). Biotin-conjugated anti-rabbit and anti-mouse antibodies (1:30 dilution, Dako Corporation, Carpinteria, California, United States) were used as secondary antibodies for avidin-biotin amplified 3,3′-diaminobenzidine staining as well as fluorescein isothiocyanate (FITC) and Texas Red conjugated secondary antibodies for cryosection immunostaining. To specifically localize BGT-1, AQP2, and alpha-1-Na-K ATPase, cryosections were tested with anti-Uromodulin-Tamm-Horsfall that labels the diluting segment (medullary thick ascending limbs of Henle’s loop, m-TAL) and not the collecting duct.

### Relationship Between Urine and Renal Medullary AQP2: Effects of Low and High Water Intake

**FIGURE 1:**
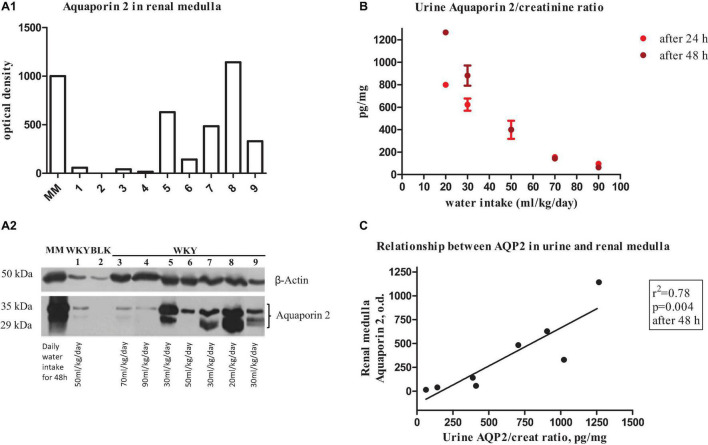
**(A1)** Bar graphs show quantitative changes in renal medulla aquaporin 2 protein expression in young female WKY rat measured by optical densitometry of immunoblotting (below). Bars represent the sum of the optical densities of the two bands observed for aquaporin 2 water channel (lower and upper bands represent native and glycosylated aquaporin 2, respectively). **(A2)** Representative immunoblot of AQP2 in renal medulla of a female WKY. Blots were incubated with polyclonal anti-AQP2 and β-Actin antibodies. MM indicates molecular weight markers. Lane 1, 6: daily water intake of 50 ml/kg; lane 3: daily water intake of 70 ml/kg; lane 4: daily water intake of 90 ml/kg; lanes 5, 7, 9: daily water intake of 30 ml/kg; and lane 8: daily water intake of 20 ml/kg. BLK indicates blank without primary anti-AQP2 antibody. **(B)** Urine aquaporin 2/creatinine ratio at different water intake (from 20 ml/kg to 90 ml/kg at 24 h and 48 h). **(C)** Relationship between urine aquaporin 2/creatinine ratio and renal medulla aquaporin 2 protein expression in the young female WKY rat.

A separate pilot experiment was performed to evaluate the relationship between AQP2 renal medullary expression and urine AQP2/creatinine ratio during low and high water intake. A small group of young female WKY (*n* = 8) had a prolongation of metabolic cage stay for 48 h when they received a low water intake (water to drink limited to 20 or 30 ml/kg/day, *n* = 4) after the 3 days of urine collection by protocol, or a high water intake (water to drink limited to 70 or 90 ml/kg/day, rat free to drink and the difference to reach the established amount by gavage, *n* = 2) compared to normal water intake (50 ml/kg/day, *n* = 2). Rats had measurements of urine output at 24 and 48 h and were rapidly euthanized with diethyl ether. Both kidneys were rapidly excised, washed with cold phosphate buffer saline, and the cortex and medulla areas were separated and immediately frozen in liquid nitrogen for immunoblotting analysis. Frozen samples were homogenized (Ultraturrax T8, Janke and Kunke, Staufen, Germany) and further sonicated (four times at 4°C for 30 s) in the extraction buffer (pH 7.4) containing 138 mM NaCl, 1.5 mM KH_2_PO_4_, 2.7 mM KCl, 0.05% Triton X-100, 2 mmol/L protease inhibitor solution [4-(2-aminoethyl) benzenesulphonyl fluoride (AEBSF), 4 μmol/L aprotinin, 0.1 mmol/L leupeptin, 200 μmol/L bestatin, 50 μmol/L pepstatin A, 15 μmol/L E-64], 0.44 mmol/L O-phenanthroline, 1 mmol/L EGTA, and 0.1% sodium dodecyl sulfate. Homogenates were centrifuged at 12,000 rpm for 10 min at 4°C. The protein concentration of the supernatant was determined using the Bio-Rad protein assay (Bio-Rad, Hercules, California, United States). Renal medulla lysates were subjected to standard SDS-PAGE and Western blotting techniques using 5% for stacking and 8–12% polyacrylamide separating gel. Equal amounts of proteins (75 μg/well) were loaded. Separated proteins were transferred to polyvinylidene difluoride (PVDF) membranes (Immun-Blot PVDF Membrane, Bio-Rad Laboratories, Hercules, California, United States) at 4 mA/cm^2^ for 45 min. The membranes were then incubated with primary antibodies anti-AQP2 (Anti-Aquaporin 2-phospho S264, 1:1,500 dilution, ab254071) and anti β-actin (Anti-β Actin, 1:4,000 dilution, ab115777, Abcam, Cambridge Biomedical Campus, Cambridge, United Kingdom), and the signal was amplified using an Opti-4CN Detection Kit (goat anti-rabbit and anti-mouse HRP-conjugated secondary antibodies, Bio-Rad Laboratories, Hercules, California, United States). Medullary expression of AQP2 (Figures 1A1, A2), urine AQP2/creatinine ratio (Figure 1B), and their relationship at different amounts of water intake in young female WKY (Figure 1C) indicate a good level of correlation (determination coefficient *r*^2^ = 0.78, *p* = 0.004, *n* = 8) suggesting that measurement of urine AQP2/creatinine ratio could be a valuable marker to evaluate water balance during the lifetime course in these rats.

### Time Course of SHR and WKY: From the Prehypertensive Phase to Organ Damage

At the end of the prehypertensive phase experiments, as indicated above, the remaining rats (30 SHR and 15 WKY) were periodically evaluated for body weight and hemodynamic parameters (BP and heart rate) at 8, 11, 15, 18, 21 24, and 27 weeks of age. At the age of 14–15 weeks, rats from both groups underwent a 3-day metabolic stay to measure urine albumin, electrolytes, creatinine, osmolality, and AQP2. Finally, at the age of 28–30 weeks, SHR and WKY repeated the same procedures performed in the prehypertensive phase with the euthanasia of all the rats following the metabolic cage stay period.

### Second Protocol: Effect of Active Treatment With ADH V1 and V2 Receptor Antagonists

In the second set of experiments, after a few days of acclimatization, a group of 28 female 25-day-old (3.5 weeks of age) SHR (Charles River France L’Arbresle, France) were divided into three groups. (i) Untreated SHR (vehicle by gavage) (*n* = 8), (ii) treated from the 25th to 49th day of age with the selective ADH V1 receptor antagonist (OPC 21268-treated SHR, *n* = 10, N-[3-[4-[4-(2-oxo-3,4-dihydroquinolin-1-yl)piperidine-1-carbonyl]phenoxy]propyl]acetamide, 5 mg/kg/day by gavage, 3924/10, Tocris Bioscience, Bristol, United Kingdom), and (iii) treated from the 25th to 49th day of age with selective ADH V2 receptor antagonist (OPC 41061-treated SHR, *n* = 10, N-[4-[(7-Chloro-2,3,4,5-tetrahydro-5-hydroxy-1H-1-benzazepin-1-yl)carbonyl]-3-methylphenyl]-2-methylbenzamide, 0.5 mg/kg/day by gavage, Otsuka Pharmaceutical, Osaka, Japan). A group of 12 female untreated WKY rats were also used as controls. All these rats had their tail-cuff systolic BP monitored non-invasively every week starting from the 3rd week of age before the initiation of the active drug treatment period until the 8 weeks of age and then with an interval of 10 days from the 8th to 27th week of age. All of these rats were subjected four times to a 3-day metabolic cage stay as described above (period 1: 23–25 days of age before the initiation of active drug treatment; period 2: 45–49 days of age just before the end of active drug treatment, period 3: 96–106 days of age stable hypertensive phase in untreated SHR; and period 4: 168–186 days of age stable hypertensive phase and organ damage in untreated SHR).

### Drugs

All drugs, except those specifically indicated in the text, and chemical components of solutions were of analytical grade and were purchased from Sigma-Aldrich Chemical (St. Louis, MO, United States).

### Statistics

All values are expressed as means ± SD. The statistical comparison of results was done by one-factor repeated measures analysis of variance or by Student’s paired or unpaired *t*-test when appropriate. A Bonferroni *post hoc* analysis was used when appropriate. Relationships between urine AQP2/creatinine ratio and renal medulla AQP2 expression, and urine osmolality and m-TAL BGT-1 were analyzed by linear regression analysis using Pearson or Spearman correlation coefficients and reported as determination coefficient. *P* < 0.05 was considered to be statistically significant.

## Results

### Body Weight, Blood Pressure, Plasma, and Urine Parameters in Prehypertension

**FIGURE 2:**
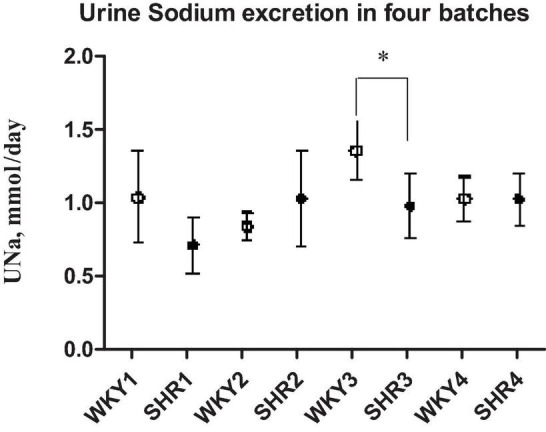
Daily sodium urine excretion in four different batches of prehypertensive 3–4-week-old female SHR and age-matched WKY. Data are expressed as means ± S.D. *p < 0.05 between SHR and WKY (batch 3). No difference in daily UNa excretion between the two strains in the other batches.

Table 1 indicates that young 3–5-week-old prehypertensive female SHRs and age-matched WKYs have similar body weight and systolic BP. No differences were measured in food and water intake at this age (Table 1). Plasma creatinine level and creatinine clearance were comparable between the two strains, as were plasma levels of sodium, potassium, and osmolality. Daily urine volume was significantly reduced in young SHR as compared with WKY (–45%, *P* < 0.05, Table 1), associated with a marked increase in urinary osmolality (*P* < 0.01, Table 1) indicating an increased urinary concentration. There was no significant difference in 24-h urinary sodium excretion, even if a mild trend toward a reduced sodium excretion was observed in young SHR; fractional excretion of sodium (FENa) was not changed. All these observations were consistent in the various batches of young SHR and WKY obtained from different colonies except for the batch 3, where a significant daily urinary sodium excretion was significantly reduced in SHR when compared to WKY (Figure 2). Daily excretion of albumin was similar between the two strains at this young age.

**TABLE 1:** Baseline parameters of the studied groups of female (25–32 days–3–5-week-old) WKY, *n* = 46 and SHR, *n* = 58.

Parameter	WKY	SHR	*P*
Body weight, g	105 ± 11	102 ± 8	NS
Systolic blood pressure, mm Hg	103 ± 8	106 ± 10	NS
Food Intake, g/day	15.1 ± 2.2	15.3 ± 1.9	NS
Water Intake, ml/day	14.2 ± 2.2	13.9 ± 2.5	NS
Urine Output, ml/day	8.8 ± 2.7	4.6 ± 1.2	*P* < 0.05
p-Creatinine, mg/dl	0.30 ± 0.04	0,32 ± 0.05	NS
Clearance Creatinine, ml/min	0.91 ± 0.10	0.88 ± 0.13	NS
p-Na, mmol/l	142.8 ± 3.3	139.8 ± 3.2	NS
p-K, mmol/l	4.7 ± 0.4	4.4 ± 0.6	NS
p-Osm, mosmol/Kg H2O	299.5 ± 6.5	300.4 ± 7.2	NS
u-Osm, mosmol/Kg H2O	1373 ± 292	2,230 ± 492	*P* < 0.01
u-Na, mmol/day	1.09 ± 0.14	0.99 ± 0.12	NS
FENa,%	0.64 ± 0.12	0.60 ± 0.08	NS
p-ADH (a), pg/ml	2.68 ± 1.10	3.15 ± 1.29	NS
u-Albumin, mg/day	0.99 ± 0.37	1.06 ± 0.48	NS
u-Aquaporin 2/Creatinine, pg/mg	410 ± 143	1,077 ± 291	*P* < 0.01
m-TAL BGT-1(a), f.u.	90 ± 48	199 ± 50	*P* < 0.01

*Values are expressed as mean ± S.D. WKY, Wistar-Kyoto rats; SHR, spontaneously hypertensive rats; Osm, osmolality; FENa, Fractional excretion of sodium; ADH, Antidiuretic Hormone, p- plasma, u- urine, Na, sodium, K, potassium; m-TAL BGT-1 medullary thick ascending limbs of Henle’s loop betaine γ-amino-n-butyric acid transporter-1; f.u., fluorescence unit; (a) the value was measured from 21 WKY rats and 23 SHR rats per group.*

### Plasma ADH, Urine AQP2, Renal BGT-1, and AQP2 Expression

**FIGURE 3:**
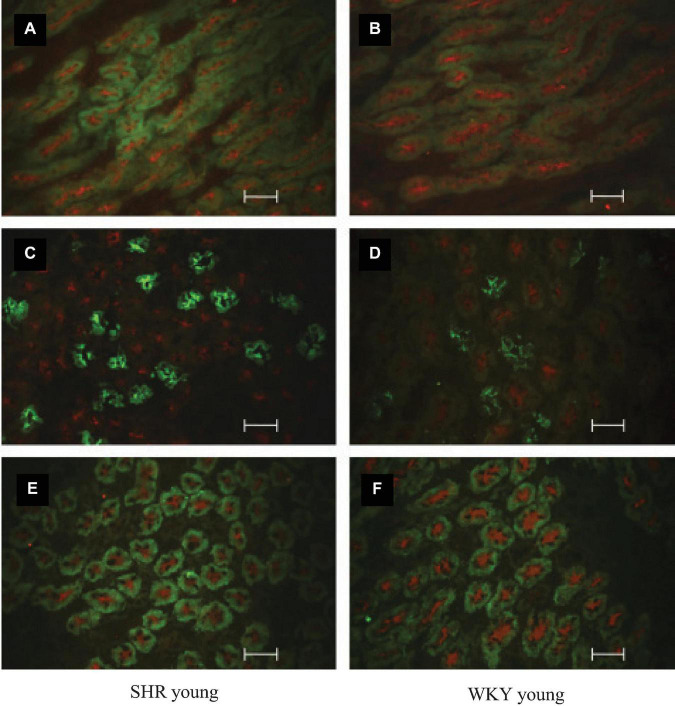
Co-immunostaining and localization of BGT-1, Uromodulin-Tamm-Horsfall, AQP2, and alpha-1-Na-K ATPase in the renal medulla of prehypertensive young female SHR vs. age-matched WKY by indirect immunostaining reaction in cryosection. Tubule BGT-1 and Uromodulin-Tamm-Horsfall co-immunostaining in prehypertensive SHR **(A)** vs. age-matched WKY **(B)**. Tubule AQP2 and Uromodulin-Tamm-Horsfall co-immunostaining in prehypertensive SHR **(C)** vs. age-matched WKY **(D)**. Tubule alpha-1-Na-K ATPase and Uromodulin-Tamm-Horsfall co-immunostaining in prehypertensive SHR **(E)** vs. age-matched WKY **(F)**. Unit: 50 μm.

**FIGURE 4:**
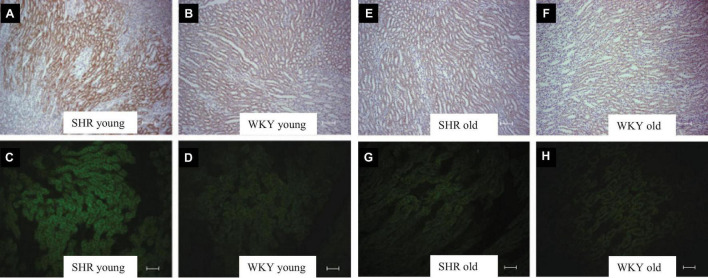
Kidney tubule BGT-1 immunostaining in prehypertensive young female SHR **(A,C)** vs. age-matched WKY **(B,D)** and in old (28–30 weeks of age) hypertensive female SHR **(E,G)** vs. age-matched WKY **(F,H)**. Indirect immunoperoxidase in paraffin sections **(A,B,E,F)** and indirect immunofluorescence reaction in cryosections **(C,D,F,H)** in young and old female SHR and WKY. Units: 200 μm **(A,B,E,F)**; 100 μm **(C,D,G,H)**.

**FIGURE 5:**
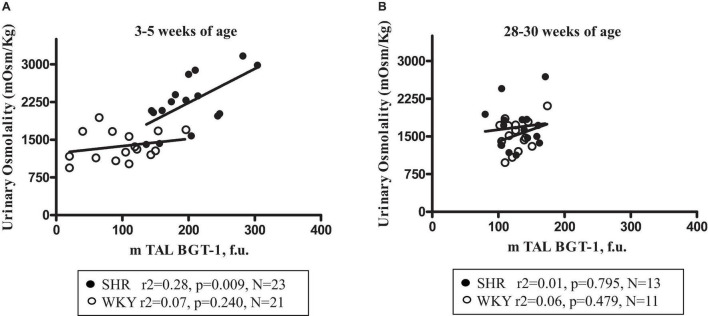
Relationship between urine osmolality and m-TAL BGT-1 expression (medullary thick ascending limbs of Henle’s loop betaine γ-amino-n-butyric acid transporter-1) in young (3–5-week-old) female SHR (filled circles) and age-matched WKY (open circles) **(A)** and in old (28–30-week-old) SHR (filled circles) and age-matched WKY (open circles) **(B)**.

Plasma ADH was not different between young SHR and WKY, but a marked rise was observed in urine AQP2/creatinine ratio in young SHR (+ 163% vs. young WKY, *P* < 0.01, Table 1). Young SHR also showed a parallel increase in immunostaining for AQP2-phospho-serine-256 in the collecting duct (identified by the absent colocalization with Tamm-Horsfall) (Figure 3C) vs. WKY (Figure 3D). Another interesting observation indicating an increased tonicity of the medullary interstitium in young SHR (Figures 3A, 4A,C) as compared to WKY (Figures 3B, 4B,D) is represented by a renal medulla increase in immunostaining (by immunoperoxidase and immunofluorescence) for m-TAL BGT-1 (+ 121% vs. WKY, Table 1) that colocalizes with Tamm-Horsfall protein. In young SHR, BGT-1 expression in m-TAL was directly related to urinary osmolality (*r*^2^ = 0.28, *P* = 0.009, *n* = 23, Figure 5A) and to AQP2/creatinine ratio (*r*^2^ = 0.19, *P* = 0.038, *n* = 23). No significant relationship was observed in young WKY between tissue m-TAL BGT-1 and urinary osmolality (*r*^2^ = 0.07, *P* = 0.240, *n* = 21, Figure 5A) or AQP2/creatinine ratio (*r*^2^ = 0.02, *P* = 0.494, *n* = 21). A mild increase of alpha-1-Na-K ATPase immunostaining in m-TAL (by immunofluorescence) that colocalizes with Tamm-Horsfall protein was observed in young SHR (+ 48%) vs. age-matched WKY (Figures 3E,F). All these results can support an increased sensitivity to ADH in this prehypertensive phase in female SHR.

### Old Hypertensive SHR With Organ Damage

Table 2 indicates that 28–30-week-old female SHRs showed, as expected, higher systolic BP levels but lower body weight vs. age-matched WKYs. No differences were measured in food and water intake at this age (Table 2). When compared to age-matched WKY, SHR had higher plasma creatinine levels and lower creatinine clearance, indicating the development of mild reduction in renal function; sodium, potassium and plasma osmolality were similar (Table 2). Daily urine volume was mildly but significantly reduced in old SHR as compared with WKY (–23%, *P* < 0.05, Table 2), without differences in urinary osmolality (Table 1). There was no significant difference in 24-h urinary sodium excretion between the two strains at this age. Daily excretion of albumin was increased in old SHR (+ 70%, *P* < 0.01) vs. age-matched WKY. Plasma ADH was higher in old vs. young SHR but also increased as compared with old WKY. There were no differences in urine AQP2/creatinine ratio between old SHR and WKY; urine AQP2/creatinine ratio was significantly lower in old SHR vs. young SHR. No difference between old SHR and WKY in tubule immunostaining (by immunoperoxidase and immunofluorescence) for m-TAL BGT-1 (Table 2 and Figures 4E–H). In old SHR, BGT-1 expression in m-TAL was not correlated with urinary osmolality (*r*^2^ = 0.01, *P* = 0.795, *n* = 13, Figure 5B) or AQP2/creatinine ratio (*r*^2^ = 0.03, *P* = 0.553, *n* = 13). No significant relationship was observed in the old WKY between tissue m-TAL BGT-1 and urinary osmolality or AQP2/creatinine ratio. No difference was observed in alpha-1-Na-K ATPase immunostaining in m-TAL or in collecting duct AQP2-phospho-serine-256 in old SHR vs. age-matched WKY. All these results indicate a reduced renal tubule sensitivity to ADH in the hypertensive phase of female SHR with organ damage.

**TABLE 2:** Baseline parameters of the studied groups of female (195–211 days–28–30-week-old) WKY, *n* = 15 and SHR, *n* = 30.

Parameter	WKY	SHR	*P*
Body weight, g	262 ± 35	229 ± 22	*P* < 0.01
Systolic blood pressure, mm Hg	118 ± 13	189 ± 14	*P* < 0.01
Food Intake, g/day	21.1 ± 1.6	20.8 ± 2.1	NS
Water Intake, ml/day	28.3 ± 3.9	24.6 ± 6.0	NS
Urine Output, ml/day	11.1 ± 0.7	8.6 ± 0.5	*P* < 0.05
p-Creatinine, mg/dl	0.35 ± 0.08	0.56 ± 0.10	*P* < 0.01
Clearance Creatinine, ml/min	1.70 ± 0.44	1.20 ± 0.48	*P* < 0.05
p-Na, mmol/l	142.6 ± 5.5	140.6 ± 5.1	NS
p-K, mmol/l	4.6 ± 0.5	4.7 ± 0.6	NS
p-Osm, mosmol/Kg H2O	297.3 ± 6.1	298.5 ± 7.0	NS
u-Osm, mosmol/Kg H2O	1,527 ± 306	1,683 ± 427	NS
u-Na, mmol/day	1.68 ± 0.26	1.58 ± 0.27	NS
FENa,%	0.54 ± 0.10	0.60 ± 0.09	NS
p-ADH (a), pg/ml	2.78 ± 1.51	8.95 ± 4.11	*P* < 0.01
u-Albumin, mg/day	1.45 ± 0.66	2.46 ± 1.20	*P* < 0.01
u-Aquaporin 2/Creatinine, pg/mg	433 ± 137	594 ± 229	NS
m-TAL BGT-1(a), f.u.	127 ± 27	129 ± 26	NS

*Values are expressed as mean ± S.D. WKY, Wistar-Kyoto rats; SHR, spontaneously hypertensive rats; Osm, osmolality; FENa, Fractional excretion of sodium; ADH, Antidiuretic Hormone, p- plasma, u- urine, Na, sodium, K, potassium; m-TAL BGT-1 medullary thick ascending limbs of Henle’s loop betaine γ-amino-n-butyric acid transporter-1; f.u., fluorescence unit; (a) the value was measured from 11 WKY rats and 13 SHR rats per group.*

### Very Early Treatment Effect of ADH V1 and V2 Receptor Antagonism on Blood Pressure

**FIGURE 6:**
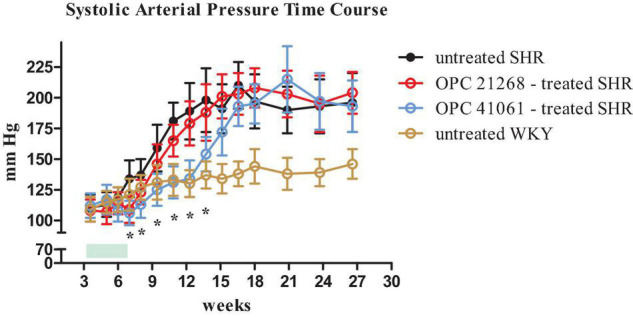
Time course of systolic arterial pressure from 3 weeks (prehypertensive phase) until 27 weeks of age in four groups of female rats: untreated SHR (*n* = 8, filled black circle), OPC 21268-treated SHR (*n* = 10, open red circle), OPC 41061-treated SHR (*n* = 10, open blue circle), and untreated WKY (*n* = 12, open yellow circle). Green rectangle indicates the active period of drug treatment (24 days). Data are expressed as means ± SD. *p < 0.01.

**FIGURE 7:**
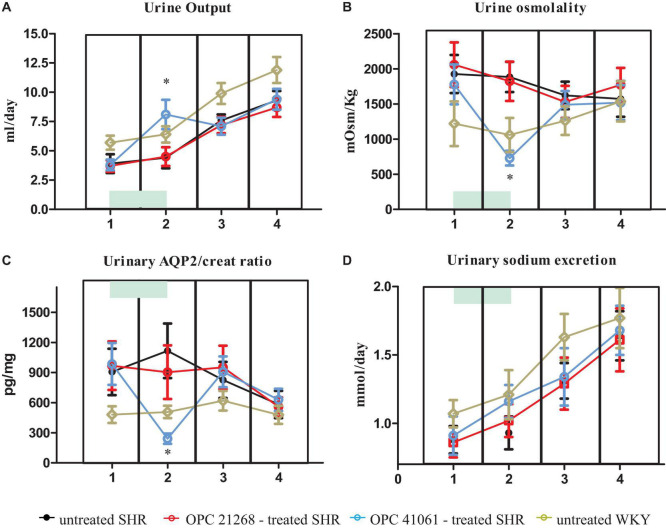
Daily urine output **(A)**, urine osmolality **(B)**, urine aquaporin 2/creatinine ratio **(C)**, daily UNa **(D)** in untreated SHR (*n* = 8, filled black circle), OPC 21268-treated SHR (*n* = 10, open red circle), OPC 41061-treated SHR (*n* = 10, open blue circle), and untreated WKY (*n* = 12, open yellow circle) at 23–25 days of age (period 1: prehypertensive phase, before the initiation of active drug treatment), at 45–49 days of age (period 2: just before the end of active drug treatment), at 96–106 days of age (period 3: stable hypertensive phase in untreated SHR); and at 168–186 days of age (period 4: stable hypertensive phase and organ damage in untreated SHR). Data are expressed as means ± SD. *p < 0.01 of OPC 41061-treated SHR vs. untreated SHR.

Figure 6 depicts the systolic BP profile from the prehypertensive phase up to the 27th week of age of the four studied groups: untreated SHR, OPC 21268-treated SHR, OPC 41061-treated SHR, and untreated WKY. ANOVA analysis of the systolic BP time course revealed an increased BP over time (*P* < 0.001), with a significant effect of the treatment (*P* < 0.01) and also a significant interaction between the time and the treatment (*P* < 0.001) (Figure 6). In untreated female SHRs, systolic BP levels rose around the 7th to 8th week and reached a plateau around the 13th to 14th week of age. In SHRs, the early active treatment with OPC 21268 slightly (but not significantly) decreased systolic arterial pressure, but it did not interfere with the future rise of BP after its withdrawal. On the contrary, OPC 41061 instead showed an attenuation of the rise in systolic BP, delaying the development of hypertension by 5.5 weeks (Figure 6). As expected, an increased volume of hypotonic urine observed during the active treatment in OPC 41061-treated SHR compared with untreated SHR and OPC 21268-treated SHR (*P* < 0.01, Figures 7A,B). No significant differences were observed in 24-h urinary sodium excretion rate among OPC 21268-treated or OPC 41061-treated SHR and untreated SHR (Figure 7D). Urine AQP2/creatinine ratio was markedly reduced by active treatment with OPC 41061 indicating a profound decrease in AQP2 shedding from apical tubule membrane into the urine (Figure 7C).

## Discussion

In this study, we demonstrated an early involvement of the increased activity of the renal ADH pathway among the mechanisms responsible of arterial hypertension development in female prehypertensive SHRs. By combining the observations of the first and the second series of experiments in prehypertensive young SHRs, an altered water balance due to an increased renal sensitivity to ADH was clearly evident in comparison to age-matched WKY; such an observation is associated with the effectiveness in preventing the future BP rise in young SHR of the early ADH V2 receptor antagonism of OPC 41061 treatment (from the 25th to 49th day), even after drug withdrawal. These results emphasize the importance of the positive water balance in this early but normotensive phase of female SHRs where a reduced daily urine volume with high osmolality represents a clinical feature, already reported in the literature in 3- and 10-week-old male SHR (57, 63). In prehypertensive female SHRs compared to age-matched WKY, ADH circulating levels were similar, but only in the former group, a strong activation of renal water absorption mechanisms was evident. The increased urine concentration was associated with the observation of a hypertonic medullary interstitium in young prehypertensive female SHRs, evidenced by the increased tubule m-TAL BGT-1 immunostaining (which colocalizes with Tamm-Horsfall protein, identifying the water impermeable tubule segment) and by a higher AQP2-phospho-serine-256 protein expression in collecting duct (where Tamm-Horsfall protein was not expressed), and by the urine AQP2/creatinine ratio. The abundance of m-TAL BGT-1 expression showed a positive correlation with urine osmolality and AQP2/creatinine ratio, further supporting the increased medullary hypertonicity in early prehypertensive SHR that is crucial for water conservation. These results were consistent over different batches and colonies of SHRs included in our study. Our results in young prehypertensive SHRs clearly support the increased renal sensitivity to ADH involving V2 receptor stimulation and finally leading to water conservation. The complex cascade of renal events following ADH binding to the G-protein–coupled V2 receptor consists of multiple steps, including cAMP-regulated protein kinase A signal transduction leads to a series of serine phosphorylations of AQP2 that allows water channel translocation from intracellular vesicles to the apical plasma membrane (13, 14, 21). The increase in urine AQP2 excretion (and the AQP2/creatinine ratio) in prehypertensive female SHRs is the result of the shedding phenomenon from the apical luminal plasma membrane of the collecting duct, where the AQP2-phospho-serine-256 protein is highly expressed as compared to young WKY. The carboxyl terminus of AQP2 harbors several phosphorylation sites (at least four sites in the rat, i.e., Ser256, Ser261, Ser264, and Ser269) that, *via* interaction with accessory proteins, are fundamental for AQP2 trafficking to the luminal plasma membrane, AQP2 apical retention, ubiquitylation, and proteasomal degradation by endocytosis (14, 21, 64, 65). In our study, we used two types of antibodies against AQP2 phosphorylated at serine S256 and S264 (anti-Aquaporin2-phospho S256 for tissue immunofluorescence studies and anti-Aquaporin 2-phospho S264 for the immunoblotting evaluation), both associated with trafficking of AQP2 to the apical plasma membrane, but with the former phosphorylation at Ser-256 representing the first and essential step for ADH-mediated AQP2 apical translocation and accumulation related to reduction of endocytosis and degradation (66–69). Despite the observation of the marked increase of AQP2-phospho-serine-256 protein in the collecting duct, our experiments are not complete. Considering the complexity of the mechanisms involved in the metabolism and trafficking of AQP2, it would be interesting and complementary to obtain information on AQP2 phosphorylation at serine 269 and 261. Even if AQP2 serine 256 phosphorylation is the fundamental and necessary step for apical trafficking, phosphorylation at serine 269 decreases AQP2 endocytosis (70), and dephosphorylation at serine 261 is associated with lower AQP2 ubiquitylation and proteasomal degradation (71–73). Therefore, the finding of an increased sensitivity to the action of ADH in the kidney of the young prehypertensive SHR may depend on alterations involving the phosphorylation of serine 256, as we have shown, but also the possible dephosphorylation of serine 261 or the phosphorylation of 269 should be considered. Moreover, even modifications of the signal machinery involved in AQP2 ubiquitylation (E3 ligases BRE1B, CUL5, NEDD4-1, and NEDD4-2) could lead to a reduction of AQP2 degradation *via* the proteasomal pathway, determining the rise of apical AQP2-phospho-serine-256 protein in young SHR and representing a potential mechanism responsible for the increased sensitivity to ADH (14, 73, 74). Unfortunately, from our observations, we cannot discriminate among the possible mechanisms cited above.

Another point that should be taken into account in the interpretation of our results relates to the fact that increased medulla tonicity *per se* can regulate apical AQP2 abundance by acting independently of ADH V2 receptor stimulation and protein kinase A pathways (19, 75). The direct correlation between tissue m-TAL BGT-1 immunostaining and urine AQP2/creatinine ratio could suggest this effect of hypertonicity *per se* on increased urinary shedding of AQP2 (related to higher collecting duct expression of AQP2-phospho-serine-256 protein), also considering that the circulating ADH levels were similar between young female SHR and WKY. This could suggest an independence from the effects of ADH in the kidneys of young SHRs and how the positive balance of water in the prehypertensive phase may be linked to hypertonicity. However, the effect of OPC 41061, the ADH V2 receptor antagonist therapy, in the second protocol of experiments delaying hypertension development shows the relevance of ADH in this phenomenon of hypertonicity and how in the prehypertensive phase of SHR, there is an increased sensitivity to ADH. Such an increase in interstitial tonicity of the renal outer medulla has been reported in the literature in 10–12 weeks of age already hypertensive male SHR, where the AQP2 expression was increased with prominent apical labeling in principal cells of the collecting duct (63).

Beside the transepithelial water permeability linked to the apical retention of AQP2, it should be noted that extremely important in the renal ADH action is the generation and maintenance of an interstitial medulla elevated tonicity. In fact, both the phenomena of transepithelial water transport and renal medulla hypertonicity, which create the osmotic gradient between the tubule fluid and the interstitium, which are regulated by ADH, are tightly interconnected (76). In fact, while the former needs the apical AQP2 expression as well as the basolateral AQP3 and AQP4 in the principal cell of the collecting duct, the latter needs sodium reabsorption and concentration in the renal medullary interstitium *via* the countercurrent multiplication mechanism coupled with urea accumulation leading to hypertonicity that represents the driving force for water reabsorption in the renal connecting tubule and collecting duct (14, 21). In this study, we observed in young prehypertensive female SHRs the up-regulation of alpha-1-Na-K ATPase immunolabelling in m-TAL that can contribute to the medulla interstitial hypertonicity. Our observations are unfortunately limited just to one of the transport mechanisms (basolateral alpha-1-Na-K ATPase in m TAL) potentially involved in ADH-dependent anti-natriuretic effects, whereas many other sodium transporters, including m-TAL NKCC2, thiazide-sensitive distal convoluted tubules-NCC, the collecting duct ENaC (whose activation is already described in literature), may contribute to ADH-mediated establishment and maintenance of interstitial medulla hypertonicity (19–21, 77). Therefore, our experiments cannot answer the question of which sodium transporter/s (besides the evaluated immunolabelling of alpha-1-Na-K ATPase) is/are crucial in prehypertensive young SHR in determining the medullary hypertonicity and high urine osmolality, but also cannot indicate whether or not there is an involvement of the urea transporters to increase urea permeability in the inner medulla to maintain its high interstitial concentration as a result of increased ADH renal sensitivity. ADH is, in fact, recognized to increase urea permeability in rat inner medulla collecting duct cells through the stimulation of V2 receptor/adenylyl cyclase/cAMP pathway that induces phosphorylation at serine 486 of UT-A1 transporter by both protein kinase A and exchange protein activated by cAMP (Epac) (78, 79). Besides ADH-mediated stimulation of urea transport *via* apical UT-A1 and basolateral UT-A3, hypertonicity *per se* can determine *via* a protein kinase C pathway the phosphorylation and activation of UT-A1 and UT-A3 in the inner medulla (21, 80, 81).

From our experiments, we cannot argue that the expression of the inner medulla urea transporter is a factor involved in the increased ADH sensitivity.

As described in the “Introduction” section, contrasting results in the literature have been reported on ADH circulating levels in the experimental model of SHR, with puzzling results showing higher or lower levels in young and old rats (31, 82) or a marked reduction in prehypertensive and hypertensive phases of male SHR (31, 32, 82, 83). These discrepancies, even in SHR of the same age and sex, are not always easy to explain and may be related to different methods of analysis, different experimental conditions, such as water intake, but also, especially in older studies, from the need to pool plasma samples to obtain sufficient amount of plasma for the hormone analysis.

It is also interesting to note that the absolute amount of daily UNa excretion was not significantly different (in our study, only in 1 batch over 4, a significant reduction of daily Na excretion was observed in female young SHRs). It can be speculated from these observations that water retention plays an important role and that it precedes sodium positive balance.

The increased sensitivity to ADH in young female SHRs is not maintained at the age of 28–30 weeks when old female SHRs are stably hypertensive and have developed organ damage (reduction of glomerular filtration rate with elevated urinary excretion of albumin), where no difference was observed regarding urinary osmolality, AQP2/creatinine ratio, and BGT-1 expression in the two strains. A kind of renal resistance to ADH effect seems to be observed in old female SHR, when the circulating ADH values in our study are much higher than in age-matched WKY and young prehypertensive SHR.

In this study, the second series of experiments was developed following the interesting results of the first series: the increased sensitivity to ADH in the prehypertensive phase of female SHRs led us to evaluate the effect of ADH V1 and V2 receptor antagonism. The treatment with V1 and V2 receptor antagonists for a short period of time (from the 25th to 49th day of age) in the early phases of post-weaning female SHR showed a different profile of future arterial hypertension development. In fact, OPC 41061, the ADH V2 receptor antagonists, delayed the development of hypertension for 5 weeks, whereas the increase in BP in female OPC 21268-treated SHR started at the end of active treatment. OPC 41061 increased expected urine output as well as water intake; a significant reduction in urine AQP2/creatinine ratio was also observed. All these results indicate that water retention could represent an important element involved in the development of high BP in this strain, or even clearer, how important is the window between 3 and 7 weeks of age to prevent the development of arterial hypertension with ADH V2 receptor antagonist. As described in the “ADH contribution to hypertension” section, contrasting results have been reported on the major role of ADH V1 or V2 receptor in the development and maintenance of arterial hypertension in the experimental model of SHR. It has been reported that ADH V1A receptor antagonist OPC 21268 treatment of male and female SHRs from the 6th to 10th weeks of age was able to decrease BP, keeping the hypotensive effects even after the withdrawal of the drug; administration of OPC-21268 to a group of older and already hypertensive SHRs did not lead to hypotensive effects (49). These results clearly contrast the observations of our study, where no effect was observed in the future development of hypertension in OPC 21268-treated SHR. Our treatment was started earlier and a later temporal window of treatment could have determined different results also, considering the diversified density of V1 and V2 receptors during the time course life of SHRs (83). At 10 weeks of age in male SHR, an up-regulation of AQP2 channels was found in the collecting duct leading to an increased absorption of water and circulating volume expansion, suggesting that the ADH V2 receptor antagonism can attenuate arterial hypertension development (63). It is also reported in the literature that the administration of an ADH V2 receptor antagonist OPC 31260 from the 6th to 10th weeks of age in male SHR did not lower BP, and even these values were increased after drug withdrawal (58); the authors suggested that this paradoxical effect could be due to a greater stimulation of V1a receptors caused by V2 receptor blockade. These results disagree with our findings, but several explanations can be considered. First, the different temporal windows: the administration of an ADH V2 receptor antagonist could be effective only in the earlier period when the abnormalities of water balance represent a key factor. Second, we used a different ADH V2 receptor antagonist OPC 41061, which is more selective V2 vs. V1 receptor than OPC 31260 (84, 85).

## Conclusion

It appears that ADH activation is involved in the pathogenesis of arterial hypertension in the experimental model of female SHR, in terms of increased ADH sensitivity of the renal collecting duct and water retention in the prehypertensive phase and of ADH V2 receptor interference with the mechanism of BP rise. Early ADH V2 receptor antagonism effects on BP profile clearly suggest the importance of the earlier phases in the future development of arterial hypertension and a potential diversified time course of BP in the late-life of this hypertensive model of rat. Therefore, being the SHR the most representative animal model for the development of essential human hypertension, it can be suggested that water balance represents, even before sodium abnormalities, a key element in the development of high BP and, in future studies, constitutes a potential target for therapies as we observed in our study the efficacy of an early V2 antagonism treatment.

## Data Availability Statement

The raw data supporting the conclusions of this article will be made available by the authors, without undue reservation.

## Ethics Statement

The animal study was reviewed and approved by University of Parma: Local Institutional Animal Ethics Committee.

## Author Contributions

IV, ST, GG, PC, ABor, and ACab conceived and designed the study. ST, GG, MC, SD, SM, AB, SC, ABov, and AC performed experiments with animals. ST, GG, MC, SD, and SC performed plasma and urine biochemical analysis and immunohistochemistry experiments. IV, AB, JZ, AC, BP, VC, and PC collected the data and contributed to the analysis of literature data. IV, BP, ST, AC, RR, RV, ABor, and ACab discussed the results. IV, GG, AB, BP, JZ, and ACab wrote the manuscript. All authors contributed to the manuscript and approved the submitted version.

## Conflict of Interest

The authors declare that the research was conducted in the absence of any commercial or financial relationships that could be construed as a potential conflict of interest.

## Publisher’s Note

All claims expressed in this article are solely those of the authors and do not necessarily represent those of their affiliated organizations, or those of the publisher, the editors and the reviewers. Any product that may be evaluated in this article, or claim that may be made by its manufacturer, is not guaranteed or endorsed by the publisher.
